# Revealing the Improved Catalytic Properties of Modified Graphene-like Structures

**DOI:** 10.1038/s41598-020-59130-z

**Published:** 2020-02-07

**Authors:** Ki-jeong Kim, Hyun Sung Kim, Hangil Lee

**Affiliations:** 1 Pohang Accelerator Laboratory, POSTECH, Pohang, 37673 Korea; 2 Department of Chemistry, Pukyong National University, Busan, 48513 Republic of Korea; 3 Department of Chemistry, Sookmyung Women’s University, Seoul, 04310 Republic of Korea

**Keywords:** Chemistry, Materials science

## Abstract

The surface morphology and electronic structure of hexagonal graphene onion rings (HGORs), a modified graphene structure, were investigated to confirm the possibility as an efficient catalyst when compared to graphene. After confirming the formation of HGORs with a smaller width (~4.2 μm) from scanning electron microscopy (SEM) and optical microscopy images, we compared the catalytic activities of HGORs and graphene by measuring the rate of oxidation of thiophenol using high-resolution photoemission spectroscopy (HRPES). In addition, we also assessed in 4-chlorophenol degradation and the OH radical formation with a benzoic acid to confirm the possibility for photocatalytic activities of HGORs. As a result, we confirmed that HGORs, which has an increased active site due to its three-dimensional structure formed by the reaction of graphene with hydrogen, can act as an effective catalyst. In addition, we could also realize the possibility of optical applicability by observing the 0.13 eV of band gap opening of HGORs.

## Introduction

Following the discovery of graphene, there has been a great effort to exploit its unique properties in various applications^1–3^. This is because of the unusual optical and electronic properties of graphene that can be influenced by its conformation^4,5^. This dependence on conformation can be very useful in various applications including electronic devices, catalysts, and biosensors^6–11^. Hence, research aimed at finding new electronic characteristics of graphene has attracted great attention in recent times^12–14^.

Particularly with respect to the applications of graphene-based catalysts, it is likely to be a highly efficient and a low-cost useful material. However, currently-used methods to grow graphene have fundamental limitations that are related to its two dimensional inertness. In other words, the graphene itself exhibits a low catalytic activity due to the limited number of active sites that enable the catalytic reaction^15,16^. If it is possible to increase the number of active sites (broken structure of *sp*^2^ symmetry) to improve catalytic properties by making structural changes while maintaining the intrinsic properties of graphene, an efficient graphene-based catalyst can be realized.

Recently, it has been reported that a three-dimensional (3D) graphene derivative called as hexagonal graphene onion rings (HGORs) can be synthesized by regulating the hydrogen partial pressure and it could be a candidate graphene-based catalyst material^17^. In detail, it is expected that the inherent properties of the graphene itself can be somewhat weakened by the 3D structure of the graphene derivative. On the other hand, an enhancement in reactivity is expected near the 3D structure is expected, which could increase the reactivity of the material in different ways including enhanced catalytic activity^18–20^.

To investigate this possibility as a useful catalyst, we fabricated 3D hexagonal graphene structures with shapes resembling onion rings (denoted as “hexagonal graphene onion rings”, HGORs) by *in situ* growth of graphene. Briefly, by incorporating hydrogen (H_2_) into graphene grown on a SiC(0001) surface, we successfully fabricated 3D HGORs by controlling the H_2_ partial pressure and annealing temperature. Furthermore, using scanning electron microscopy (SEM), optical microscope, Raman spectroscopy, high-resolution photoemission spectroscopy (HRPES), and angle-resolved photoemission spectroscopy (ARPES), we have compared the morphologies and electronic properties of the fabricated HGORs with those of graphene.

Following the comparison of the morphological characteristics and electronic structures of the three distinct graphene-related materials (graphene, metastable-HGORs (m-HGORs), and HGORs), we assessed their catalytic activities for the oxidation of thiophenol (TP) under 365 nm-wavelength UV light illumination *via* HRPES, because the thiol group (-SH) in TP can be oxidized to form a disulfide group (-S-S-)^21,22^. In addition, to confirm the possibility of HGORs as a photocatalyst, we also assessed the photocatalytic activities of the three tested graphene-related materials in other reactions such as 4-chlorophenol (4-CP) degradation and OH radical formation of benzoic acid (BA) in aqueous solutions, which are widely used to compare the photocatalytic properties of catalyst materials^23–26^.

## Results and Discussion

### Characterization of morphological and electronic structure

**Figure 1:**
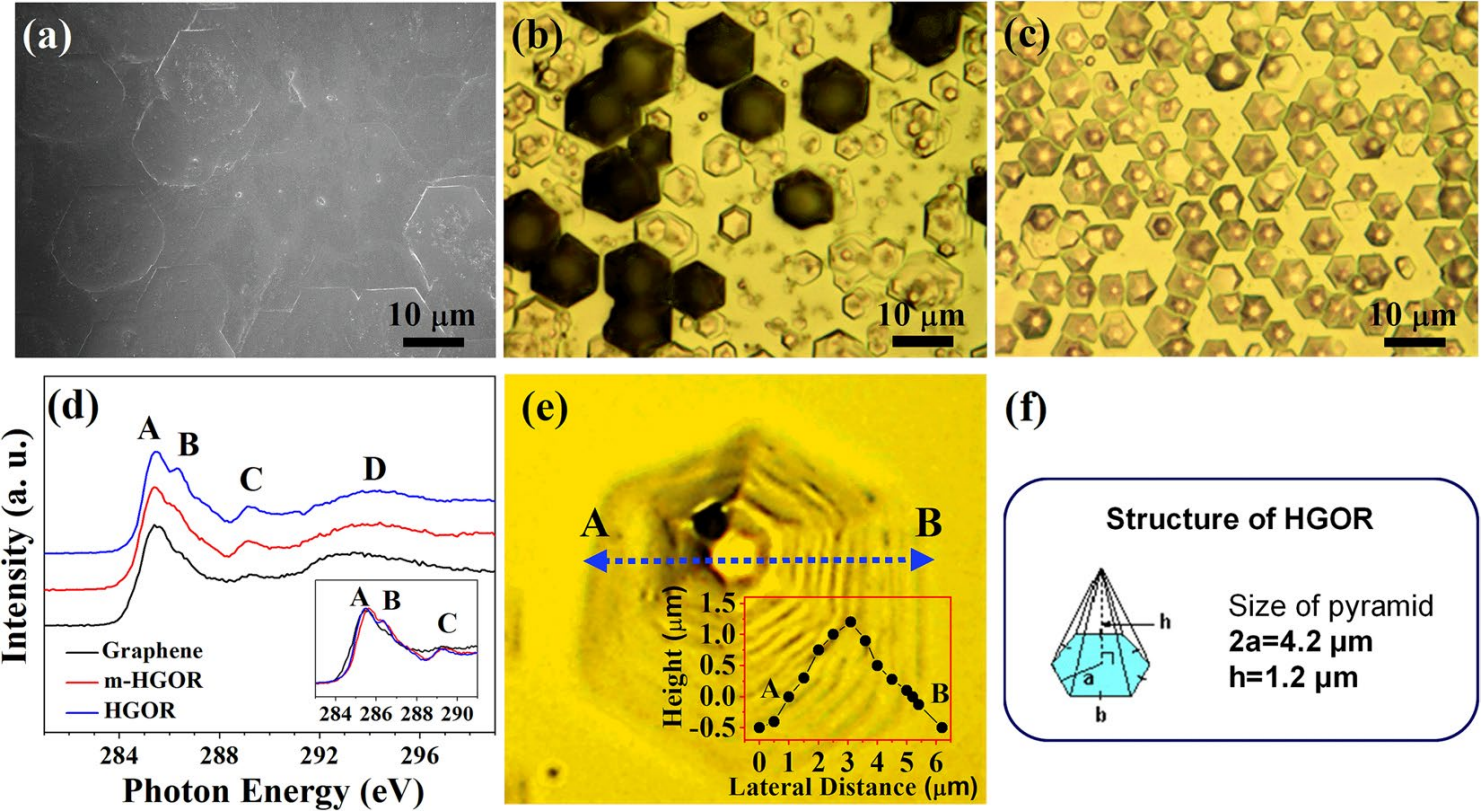
(**a**) SEM image of graphene, (**b**,**c**) optical microscopy images of m-HGORs and HGORs. (**d**) XAS of graphene sheet, m-HGORs obtained at 1650 K, and HGORs obtained at 1830 K under hydrogen gas of ~760 Torr partial pressure. (**e**) Optical microscopy image and the corresponding line profile, and (**f**) a schematic diagram of the structure of the HGORs fabricated in this work.

SEM images and optical microscope images were obtained to confirm the surface structure of graphene and graphene derivatives synthesized as described above. The morphologies of the three carbon-based materials studied in this work, i.e., graphene, m-HGORs, and HGORs, were imaged using SEM and optical microscope. As shown in Fig. 1(a), graphene sheets are obtained, similar in appearance to those reported in previous studies^11,27^. Figure 1(b,c) show optical microscope images taken after annealing at two different annealing temperatures (T_s_) (T_s_ = 1650 K and T_s_ = 1830 K) under a hydrogen gas dose of ~760 Torr. The images in Fig. 1(a–c) clearly show how HGORs are formed as the substrate annealing temperature is varied during hydrogen treatment, i.e. after forming a graphene on the substrate (Fig. 1a). At T_s_ = 1650 K (Fig. 1(b)), the graphene film appears to be fissured, while small flakes start to form (denoted as a metastable HGORs; m-HGORs). As the substrate temperature is increased to 1830 K (Fig. 1(c)), uniformly sized HGORs begin to form. As shown in the figure, HGORs were in the form of regularly-sized hexagons, and its shape was quite different from that of graphene.

Recently, Glass *et al*. illustrated in detail, the gas phase chemistry during H-passivation of graphene by scanning tunneling microscopy (STM) where desorption of a zipper-like material at the step edges was observed. Si–C sheets were removed in a layer-by-layer fashion, first leading to large terraces with straight rims^28^. And at the end of this process, an atomically smooth surface is formed. Our result is also in accordance with these observations, and in our case, we find in addition, that HGORs structures can be formed when the surface is passivated by H_2_ with partial pressure ~760 Torr as illustrated in Supplementary Material Fig. S1.

To clarify the differences in electronic structure of the three tested samples, we also performed carbon *K*-edge XAS. As shown in Fig. 1(d), a remarkable difference is observed between graphene and HGORs. A first observation reveals that the XAS spectra of the samples show typical graphene-related four peaks for all the three samples, confirmed by a sharp 1 *s* → π* peak at 285.5 eV (marked A) and by the typical line shape of the 1 *s* → σ* edge at 292.4 eV (marked D). However, a closer look reveals that the intensities of B (286.5 eV) and C (289.3 eV) peaks are higher for the HGORs structure. Hence, this difference of the electronic structure can be explained to be due to contributions from peaks induced by hydrogen^29,30^. In other words, when comparing the XAS spectra for the three tested samples, the most significant difference between graphene and HGORs is the increments of these two peaks due to hydrogen doping, while the basic electronic structure of graphene remains the same (black color).

From the optical microscope image in Fig. 1(c), we confirmed that HGORs has a hexagonal structure. To further clarify the three-dimensional (3D) structure of the HGORs, we acquired optical microscopy images of HGORs, shown in Fig. 1(e). High-magnification image reveals that the fabricated HGORs is not flat (2D) but is a 3D structure with hexagonal rings in the form of an onion. To determine the size of our HGORs, we have traced a line profile (see inset of Fig. 1(e)), from which, we measured a width of 4.2 μm and height of 1.2 μm for the HGORs. As mentioned in the introduction section, Yan *et al*. first reported well-formed and regularly shaped ~100-μm-wide HGORs grown on a Cu foil^17^. Our HGORs structure also shows a regular shape, but with a smaller width (~4.2 μm). In view of its smaller size, we expect that the HGORs fabricated in the work could be useful in practical applications. Figure 1(f) shows a schematic diagram of the growth of HGOR on a SiC(0001) substrate.

To confirm the effect of hydrogenation on the structure, the influence of H_2_ partial pressure was investigated, but no significant difference was found for partial pressures in the range 700–760 Torr. Once the optimal conditions for fabricating HGORs were determined, HRPES, Raman spectroscopy, and ARPES were performed to observe the difference in the electron structure of HGORs from that of graphene.

**Figure 2:**
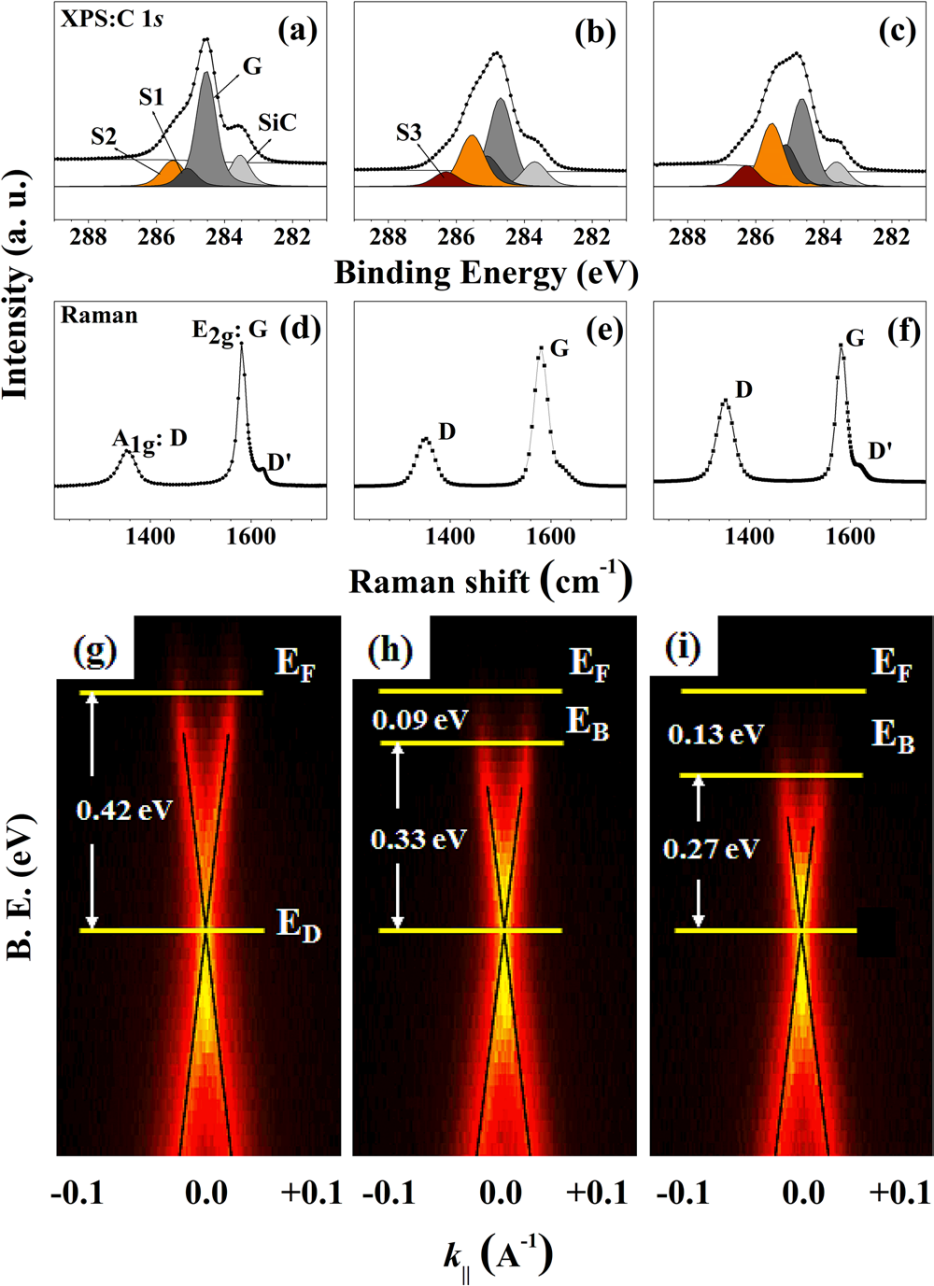
HRPES, Raman spectroscopy, and ARPES results of (**a**,**d**), and (**g**) graphene, (**b**,**e**,**h**) m-HGORs, and (**c**,f,i) HGORs at 300 K.

As shown in Supplementary Material Fig. S2, the only peak of C 1 s except Si 2 s and 2p from substrate, was observed in the XPS survey scan spectrum of the graphene and HGOR, indicating the absence of impurity in samples. We next performed electronic spectroscopic analysis using HRPES, Raman spectroscopy, and ARPES data for graphene, m-HGORs, and HGORs to elucidate the effect of hydrogen doping on the electronic structure of graphene-related materials. Figure 2(a) shows the typical C 1 *s* core-level spectra of the graphene layer grown on the SiC(0001) surface; *sp*2 carbon atoms at the interface layer (marked as S2), the 6√3 × 6√3 interface layer (buffer layer marked S1), the graphene layer (marked as G), and the SiC substrate (marked as SiC)^31,32^.

After confirming the pristine state of the graphene from C 1 *s* core-level spectra (Fig. 2(a)), we also determined the electronic structure of m-HGORs and HGORs, as shown in Fig. 2(b,c). The intensity of the G peak was observed to be lower for both m-HGORs and HGORs when compared to that for graphene. For HGORs, the G peak intensity was however, higher than those of the other C 1 *s* peaks, indicating the presence of a considerable amount of material with graphene character in HGORs, despite the morphology of HGORs being different from that of graphene. Due to increase in S2 peak which includes contributions from defect structure, a decrease in G peak can be expected following hydrogen doping. Moreover, the intensity of the S2 peak in the HGORs spectrum is higher than that in the graphene spectrum. Different from the C 1 *s* spectrum of graphene, a new peak appears for m-HGORs and HGORs, denoted by S3, which we attribute to an interface peak generated by hydrogen doping. These results confirm that hydrogen doping can be effectively used to modify the electronic structure of graphene.

Figure 2(d–f) show, respectively, the Raman spectra of graphene, m-HGORs, and HGORs grown on a SiC(0001) surface. In the Raman spectrum of graphene, peaks are observed at 1580 cm^–1^ (G peak) and 1320 cm^–1^ (D peak) resulting from the graphene layer^33,34^. In detail, comparing the intensity ratios of I_D_/ I_G_ for three samples, it can be seen that m-HGORs and HGORs have values of 0.348 and 0.642, respectively, while graphene is that of 0.218. As is well known, the increased intensity ratio is a good explanation for the 3D structure formation *via* hydrogen doping in HGORs, which means that a new interface structure has been formed, unlike graphene. In addition, since the peak at 1627.9 cm^−1^, which is indicated by D’, is the peak due to the defect induced structure of the samples and the intensity of the peak of HGORs is larger than that of graphene, it can be also explained as the difference of electronic structure between graphene and HGORs. As shown in these figures, apart from the change in intensity of the peaks due to hydrogen doping, the general characteristics of graphene are maintained for the other two samples.

For graphene, the absence of bandgap is a major disadvantage that limits its application as an optical material. In order to have semiconductor characteristics, it is necessary to have some degree of gap opening. Therefore, it is important to determine whether HGORs has a measurable band gap. To do this, we measured the difference in band gap between the three tested samples using ARPES. Figure 2(g–i) show the ARPES results of the three samples. As is well known, in the case of single-layer graphene, the energy gap can be defined as the energy difference between the Fermi level (E_F_) and the Dirac point (E_D_). As shown in Fig. 2(g), the energy value in our measurements between the Fermi level and the Dirac point of graphene is ~0.42 eV, which is in good agreement with the literature value^35,36^. Based on this result, changes in bandgap of modified m-HGORs and HGORs were measured from the ARPES spectrum, meaning that the band gap energy was estimated from the change in energy gap between the Fermi level and the Dirac point of the two samples. From Fig. 2(h,i), we clearly confirmed bandgap opening in both m-HGORs and HGORs, with band gaps of ~0.09 and 0.13 eV, respectively. This is a very interesting result, showing that a hydrogen-doped 3D structure shows the bandgap opening. From these results, we can expect that if the amount of hydrogen doping is controlled depending on the dosing time, the bandgap of HGORs can be controlled.

**Figure 3:**
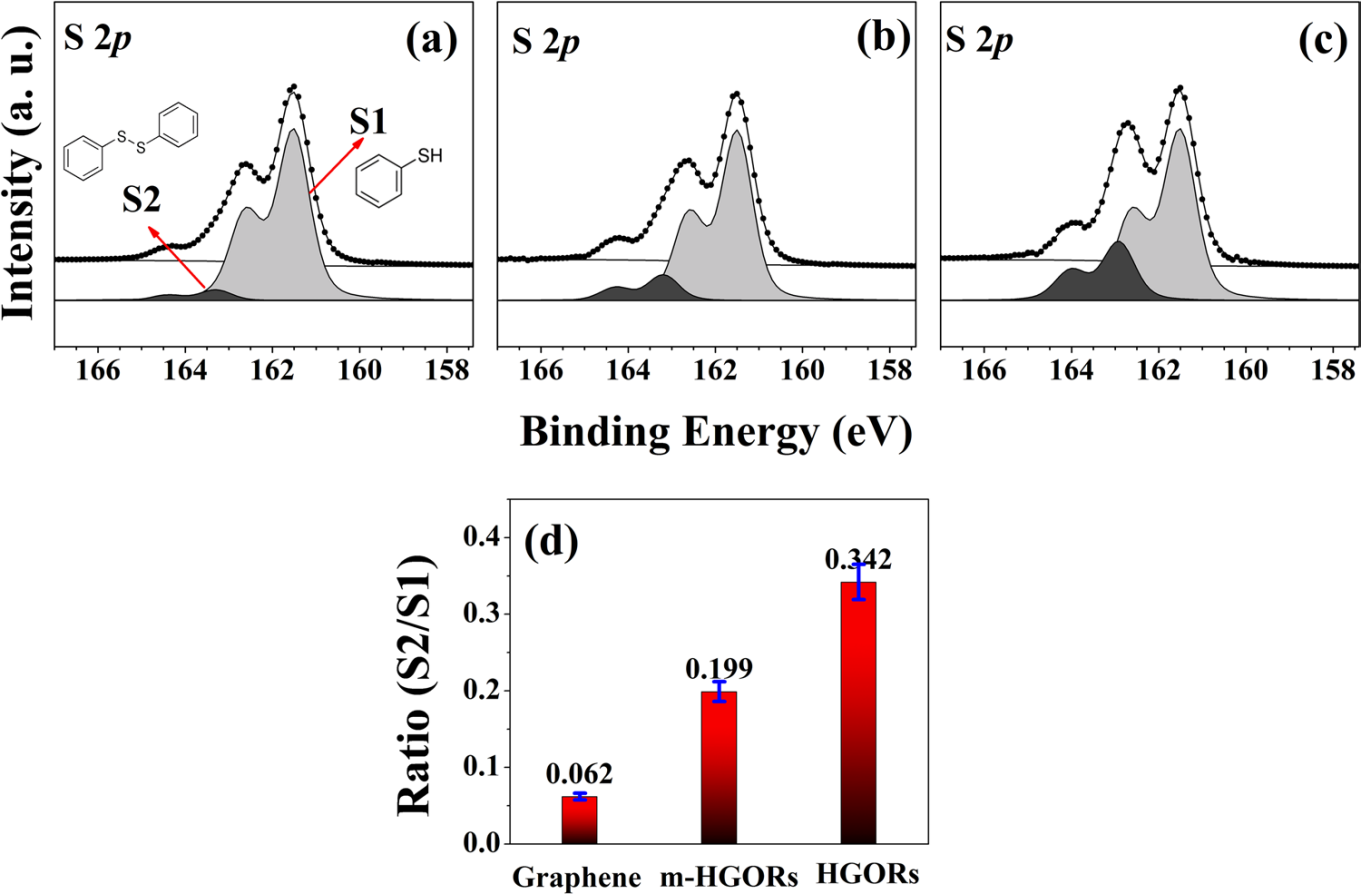
S 2*p* core level spectra taken after exposing 360 L thiophenol (TP) adsorbed on (**a**) graphene, (**b**) m-HGORs, and (**c**) HGORs, all the experiments were carried out at 300 K under 365 nm-wavelength UV light.

**Figure 4:**
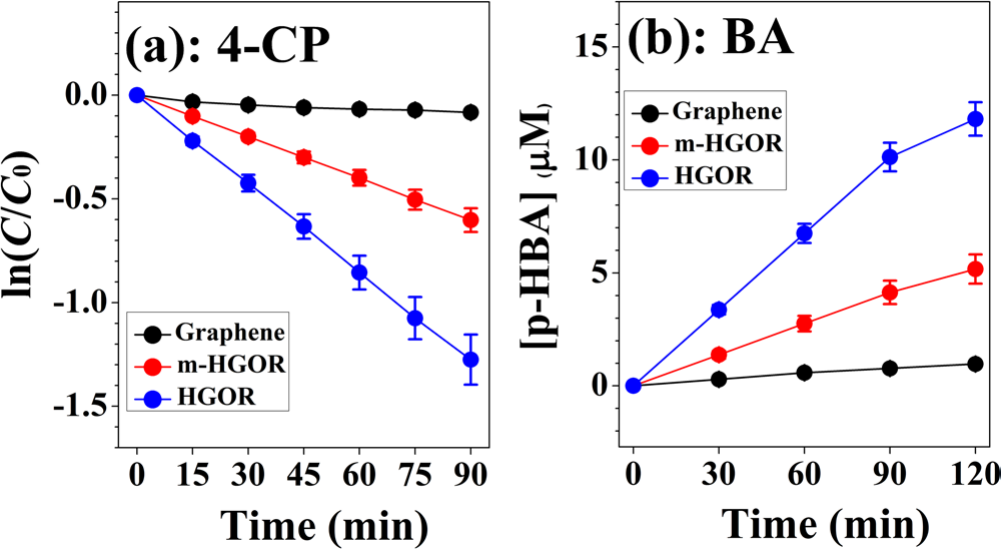
Time profiles for (**a**) 4-CP photocatalytic degradation and (**b**) radical formation of p-HBA resulting from the reaction of BA with graphene, m-HGORs, and HGORs under UV illumination conditions. λ > 320 nm and [BA]_0_ = 10 mM.

To evaluate the application potential of HGORs, we tested its possibility, which can act as a catalyst. More specifically, we assessed the catalytic activation of TP oxidation (see Fig. 3), and 4-CP degradation and OH radical formation of BA (see Fig. 4).

### Characterization of catalytic activities

First, we carried out the catalytic oxidation of 360 L TP with the same amount of molecular oxygen adsorbed on three different samples; the oxidation reactions were carried out under of 365-nm-wavelength UV irradiation. Figure 3(a–c) show the S 2*p* core level spectra of 360 L TP adsorbed on the three tested samples under UV illumination. All the spectra show two distinct S 2*p*_3/2_ peaks at 161.5 and 162.9 eV, corresponding, respectively, to the C-SH unbound state (denoted as S1) and the disulfide state (denoted as S2)^37–39^; note that the disulfide peak is formed from by the oxidation of the thiol group of TP. Because it has been shown that disulfide is an oxidation product of the thiol group [see Eq. (1)], we monitored the oxidation of TP by measuring the ratio of the intensity of S1 to that of S2 for each of the three tested samples.

1 $$2{{\rm{C}}}_{6}{{\rm{H}}}_{5}-{\rm{SH}}+\frac{1}{2}{{\rm{O}}}_{2}\mathop{\to }\limits^{hv}{{\rm{C}}}_{6}{{\rm{H}}}_{5}-{\rm{S}}-{\rm{S}}-{{\rm{C}}}_{6}{{\rm{H}}}_{5}\,+{{\rm{H}}}_{2}{\rm{O}}$$

As indicated by the rate of disulfide produced, we found that HGORs was to be more active than graphene or m-HGORs shown in Fig. 3(d). These results provide strong evidence that TP undergoes better oxidative reactions in HGORs under UV irradiation. As mentioned in the introduction, one of the most commonly used methods in a comparative analysis of the photocatalytic properties of different samples is the 4-CP degradation reaction and an efficient way to assess the photocatalytic properties is by measuring the increase in defect structure or by tracking the active site to confirm the formation of OH radicals. For this purpose, as shown in Fig. 4, we have compared the photocatalytic properties of the three samples using these two methods.

Figure 4(a) shows the 4-CP degradation curve for graphene, m-HGORs, and HGORs, where a degradation of 4-CP is observed in all the three cases. Under UV light illumination, the wavelength of light is sufficient for all the three different carbon based materials to form electron–hole pairs. Thus, HGORs effectively degrades the 4-CP solution under UV illumination after 90 min, owing to the presence of an increased number of active sites. In other words, UV light irradiation on the surface of HGORs effectively produces electrons and holes by a reduction and oxidation processes; this reaction is much faster in HGORs when compared to that on graphene or m-HGORs. In detail, plotting ln(*C/C*_0_) against time, a first order-reaction relationship was obtained. As shown in Fig. 4(a), the rates of change of HGORs are remarkably faster than that of graphene. This indicates that the photocatalytic activity of HGORs is better than that of graphene. We also calculated the rate constant value (*k*) for the change in concentration (ln(*C*/*C*_0_)) with time (min.) for the three tested samples. In detail, the apparent rate constant for the decay of the reactant for HGORs was 1.41 × 10^−2^ min^−1^ when using HGORs, faster than the cases using other tested samples (8.89 × 10^−4^ min^−1^ for graphene and 6.68 × 10^−3^ min^−1^ for m-HGORs). As mentioned above, the improvement of the catalytic properties of HGORs has previously been shown to be closely related to the 3D structure induced by hydrogen doping in he samples. Electrons and holes so produced react with aqueous 4-CP solution to generate OH radicals that are capable of effectively degrading the 4-CP solution^40^.

Our results are also consistent with the assumption that active radicals are formed on the surface of HGORs, since the reaction of OH ions with holes in HGORs has been shown to usually generate more OH radicals. To confirm the generation of a large number of OH radicals on HGORs, the radical reaction of benzoic acid was performed on these substrates. When the OH radical formed on the surface of a graphene-based sample reacts with benzoic acid (BA) as shown in Eq. (1), *p*-Hydroxyl benzoic acid (*p*-HBA) is formed. Hence, by comparing the amount of the *p*-HBA formed at a given time, the influence of the OH radical on the catalytic reaction can be analysed and compared^25,26^. Results in Fig. 4(b) confirm that the OH radical formation reaction in HGORs is significantly larger than that of the other two materials. Table 1 summarize the values of rate constants and OH radical formations of BA for the three tested samples.

**Table 1:** Values for rate constant values obtained from 4-CP degradation and the concentration of *p*-HBA obtained from BA for the three tested samples.

	graphene	m-HGORs	HGORs
*k* (×10 ^−3^ · min^−1^)	0.889	6.68	**14.0↑**
*p*-HBA(µM)	0.965	5.173	**11.813↑**

The results are also consistent with those obtained in TP oxidation and 4-CP degradation experiments. Therefore, our results proved that the 3D structure of graphene induced by hydrogen doping can enhance the catalytic activity by forming many active sites (or defect structure).

Finally, HGORs exhibiting catalytic properties must be able to maintain their properties after reuse because economic considerations must be taken into consideration in order to be applicable to various fields. Therefore, the reaction by the 4-CP degradation and the OH radical formation of BA using the HGORs, which showed good efficiency among the three tested samples. In particular, because the inertness is one of the most important features of graphene, it is very important to confirm the reuse rate for HGORs in various fields.

**Figure 5:**
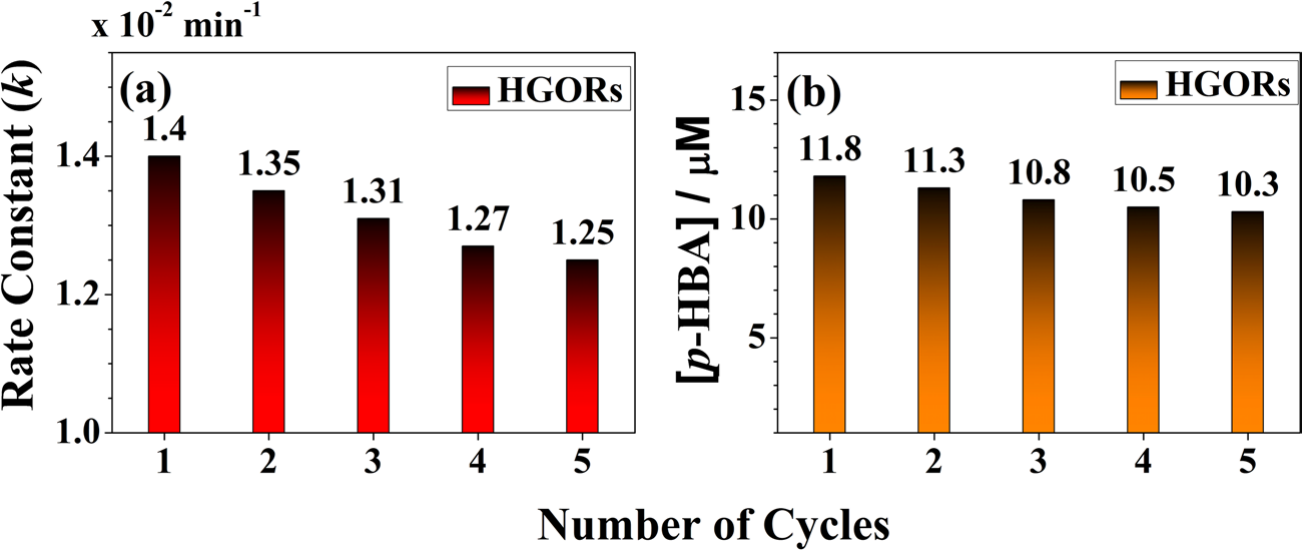
Recovery Rate for (**a**) 4-CP degradation and (**b**) OH radical formation of HGORs.

To test this, re-use experiments for 4-CP degradation and OH radical formation of the HGORs were run for 5 cycles shown in Fig. 5(a,b). As expected, the reaction rate constant obtained from 4-CP degradation and the concentration of *p*-HBA obtained from the OH radical formation of BA were reduced by only ~10.7 and 12.7% in HGORs after 5 re-use. This result was very meaningful that the HGORs shows some practical possibility, which can be applicable in the field of catalysis. Through the additional experiments, we can confirm that HGORs shows some practical applicability because it shows some degree of re-use as well as photocatalytic properties.

## Conclusion

In conclusion, we successfully fabricated HGORs with a regular size (~4.2 µm width) from graphene grown on the SiC(0001) surface by reaction with hydrogen. In order to compare the catalytic activities of graphene and HGORs in oxidation reactions, we tested the three materials in the oxidation of TP *via* HRPES. Furthermore, we also monitored the photocatalytic activity through the rate of 4-CP degradation and the concentration of OH radical formation with BA. Results of spectroscopic analyses clearly confirmed that HGORs can be potentially used as an effective catalyst with a bandgap opening (~0.13 eV) due to the creation of active sites by hydrogen doping. We further expect that the modified graphene such as HGORs will continue to be used by controlling the active sites in manufacturing the modified graphene as an efficient catalyst in the field of nanoscience.

## Methods

### Materials

Nitrogen-doped (N_D_ = 9 × 10^17^ cm^–3^) Si-terminated 6H-SiC(0001) was purchased from Cree Research (USA). Thiophenol (TP: 97% purity), 4-chlorophenol (4-CP: 97% purity), and benzoic acid (BA: 98% purity) were purchased from Sigma Aldrich (Korea).

### Fabrication of HGORs

HGORs were fabricated in the following manner: First, pristine monolayer graphene was prepared on a SiC(0001) surface. Prior to graphene formation, the temperature of the SiC(0001) surface was increased stepwise to 1120 K and left overnight for outgassing the substrate. The outgassing was completed at 1200 K and the samples were then annealed at this temperature for 5 min using a silicon dozer (1 Å/min) to obtain a Si-rich surface, the sample temperature was further increased to 1500 K and held for 2 min to form the graphene layer on the substrate. The sample was outgassed again overnight after the formation of a graphene monolayer and then placed in the preparation chamber filled with H_2_ to ~760 Torr. The sample temperature was then increased to 1830 K, when HGORs started to form on the surface. Our results show that HGORs with various sizes and shapes are formed depending on the sample annealing temperature and H_2_ partial pressure.

### Characterization

The sample morphology was analysed using field-emission SEM (FE-SEM, FEI Inspect F50, operating at 10 kV). Raman spectra data were obtained with an Ar^+^ ion laser (Spectra-Physics Stabilite 2017; λ_ex_ = 514.5 nm) excitation source and a spectrometer (Horiba Jobin Yvon TRIAX 550). HRPES, X-ray absorption spectroscopy (XAS), and angle-resolved photoemission spectroscopy (ARPES) experiments were carried out using an electron analyser (R2000, Gamma-Data Scienta.) at the 10A2 beamline (Pohang Accelerator Laboratory) to identify the electronic structure of the three tested samples. The C 1 *s* and S 2*p* core level spectra were obtained by using photon energies of 330 and 230 eV respectively to enhance the surface sensitivity. The binding energies of the core level spectra were determined with respect to the binding energies (E_B_ = 84.0 eV) of the clean Au 4 *f* core level for the same photon energy. For photocatalytic activity test, we used 4-chlorophenol (4-CP, Sigma-Aldrich, ≥99%) and benzoic acid (BA, Sigma-Aldrich, ≥99%), as target contaminants. The required amount of the three different graphene-based samples (12 mm × 2 mm in size with a thickness of 0.25 mm; 50% of HGOR population) was prepared in a reactor containing an aqueous solution of each substrate (30 mL, 100 µM). The suspensions were magnetically stirred in the dark for 20 minutes to establish absorption–desorption equilibrium. Then, the reactor was irradiated by a 300-W Xe arc lamp (Newport) with a cutoff filter for UV illumination (the wavelength was 320 nm). Solution samples including 4-CP (or BA) were collected using a 1-mL syringe every 15 min, filtered through 0.45 µm PTFE filter (Whatman), and then, analyzed. The concentrations of 4-chlorophenol and p-HBA were measured by high-performance liquid chromatography (HPLC, Shimadzu UFLC LC-20AD pump) equipped with a diode array detector and a Shim-pack GIS column (4.6 mm × 250 mm).

## Supplementary information

Supplementary Information.
