# Supplementary material for: Revealing the Improved Catalytic Properties of Modified Graphene-like Structures
Source: Sci Rep. 2020 Feb 7;10:2119. doi: 10.1038/s41598-020-59130-z (PMC7005818; doi:10.1038/s41598-020-59130-z)
Supplement: Supplementary file 1 — Supplementary Information. [file 41598_2020_59130_MOESM1_ESM.docx]

**Supplementary Information**

**Revealing the Improved Catalytic Properties of Modified Graphene-like Structures**

**Ki-jeong Kim**^1^ **Hyun Sung Kim**^*,2^ **and Hangil Lee**^*,3^

^1^Pohang Accelerator Laboratory, POSTECH, Pohang, 37673, Korea

^2^Department of Chemistry, Pukyong National University, Busan 48513, Republic of Korea

^3^Department of Chemistry, Sookmyung Women’s University, Seoul 04310, Republic of Korea


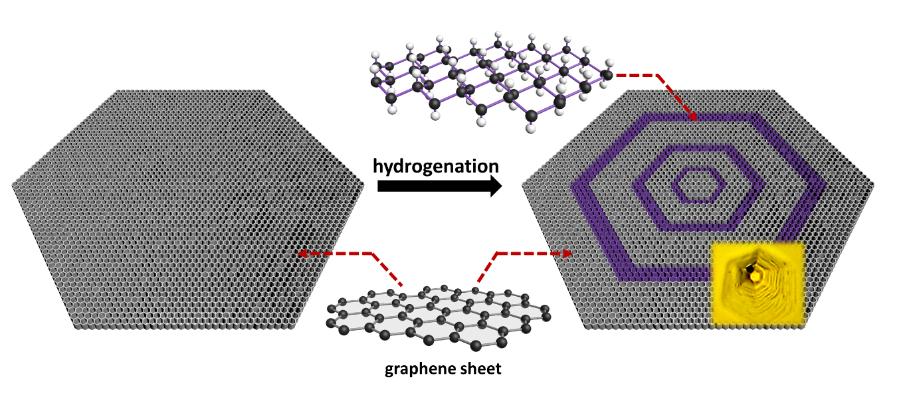
 Figure S1. Schematic diagram for change from graphene to HGOR.


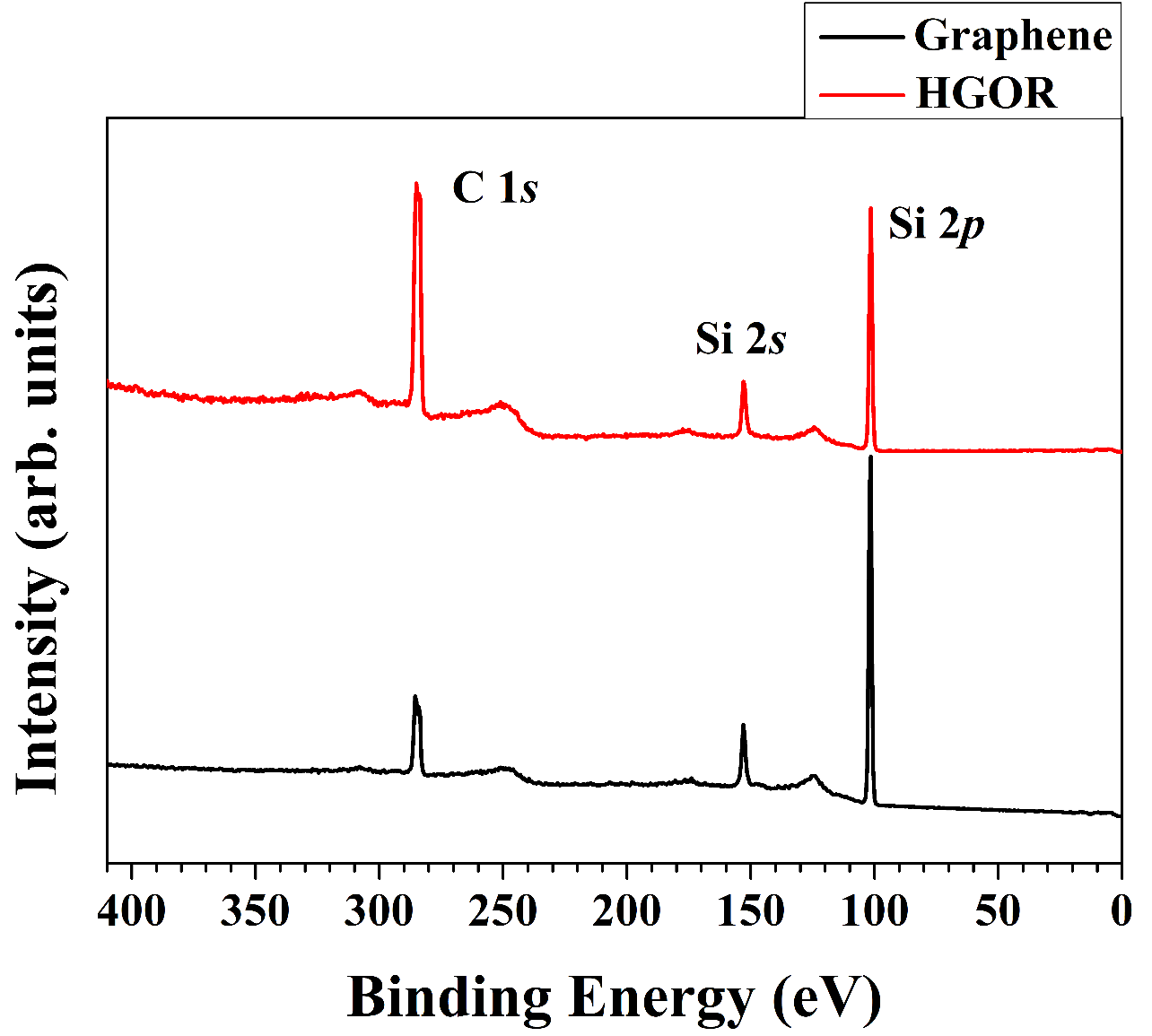


Figure S2. X-ray Photoelectron Spectroscopy (XPS) survey spectra of graphene and HGOR. The photon energy is 500 eV to enhance the surface sensitivity.
